# Influence of Hexagonal Boron Nitride on Electronic Structure of Graphene

**DOI:** 10.3390/molecules27123740

**Published:** 2022-06-10

**Authors:** Jingran Liu, Chaobo Luo, Haolin Lu, Zhongkai Huang, Guankui Long, Xiangyang Peng

**Affiliations:** 1 Hunan Key Laboratory for Micro-Nano Energy Materials and Devices, School of Physics and Optoelectronics, Xiangtan University, Xiangtan 411105, China; 201921001241@smail.xtu.edu.cn (J.L.); luochaobo@xtu.edu.cn (C.L.); 2 School of Materials Science and Engineering, National Institute for Advanced Materials, Renewable Energy Conversion and Storage Center (RECAST), Nankai University, Tianjin 300350, China; 1713638@mail.nankai.edu.cn; 3 Key Laboratory of Extraordinary Bond Engineering and Advanced Materials Technology of Chongqing, Yangtze Normal University, Chongqing 408100, China

**Keywords:** graphene, boron nitride, density of states, Fermi velocity, charge transfer

## Abstract

By performing first-principles calculations, we studied hexagonal-boron-nitride (hBN)-supported graphene, in which moiré structures are formed due to lattice mismatch or interlayer rotation. A series of graphene/hBN systems has been studied to reveal the evolution of properties with respect to different twisting angles (21.78°, 13.1°, 9.43°, 7.34°, 5.1°, and 3.48°). Although AA- and AB-stacked graphene/hBN are gapped at the Dirac point by about 50 meV, the energy gap of the moiré graphene/hBN, which is much more asymmetric, is only about several meV. Although the Dirac cone of graphene residing in the wide gap of hBN is not much affected, the calculated Fermi velocity is found to decrease with the increase in the moiré super lattice constant due to charge transfer. The periodic potential imposed by hBN modulated charge distributions in graphene, leading to the shift of graphene bands. In agreement with experiments, there are dips in the calculated density of states, which get closer and closer to the Fermi energy as the moiré lattice grows larger.

## 1. Introduction

Since graphene was successfully isolated from graphite, this two-dimensional (2D) semimetal has attracted intense attention thanks to its special features such as massless Dirac fermions, high carrier mobility, and high thermal and electron conductivity [1]. For practical applications, devices made from this fragile monocrystalline graphitic film have to be supported on substrates and were firstly fabricated on SiO_2_ substrate [2]. However, in graphene/SiO_2_ systems there are significant degradations of carrier mobilities due to charged surface states, surface roughness, and surface optical phonons in SiO_2_ [3,4]. Carrier properties can be easily changed by unclean substrate or doping in carbon-based low-dimensional materials [5,6,7]. In contrast, hexagonal boron nitride (hBN) has a 2D, flat, honeycomb network that is very similar to that of graphene, and it is identified to be a featureless dielectric substrate for graphene [8]. Being flat, inert, and wide gapped, hBN is a natural support for graphene. For instance, charge and surface fluctuations in flat hBN have been demonstrated to be several orders of magnitude smaller than those in amorphous SiO_2_ [9]. It was revealed that graphene grown on hBN is very flat, and the charge mobility is close to that of suspended graphene. Therefore, significant barriers imposed by the irregularity of other substrates can thus be removed from the way to realizations of the full functionality of graphene in electronic devices [9,10,11,12,13].

Not only have qualities of graphene been much improved by growing on hBN flakes, but also many fundamental physical phenomena have been discovered in graphene/hBN heterostructures [12,14,15,16,17,18,19,20,21,22,23,24,25,26,27,28,29,30]. For example, graphene aligned on hBN with a high-mobility exhibit integer and fractional quantum Hall effects and quantum anomalous Hall effects [19,31,32]. When the graphene and hBN crystals are rotationally aligned, a long-wavelength moiré superlattice occurs [33,34], which creates new, finite-energy Dirac points in the graphene band structure and leads to the Hofstadter butterfly spectrum [35,36]. Incommensurate-commensurate transition occurs when the spatial periodicity of the graphene/BN moiré structure is larger than 10 nm [33].

In particular, great efforts have been devoted to the clarification of the band structure modulation around the Dirac cone induced by the hBN superlattice potential [13,36,37,38,39,40,41,42,43,44,45,46]. It is reported that a band gap as large as around 40 meV emerges if graphene/hBN is nearly 0° aligned, and the gap varies with respect to the twisting angle between the two layers [18,36,38,39,42,45,46,47,48]. In addition, Fermi velocity is one of the key concepts in graphene research, as it bears information on a variety of fundamental properties in the study of graphene’s energy dispersion. It was found that the Fermi velocity near the Dirac point is reduced with the decrease in the twisting angle [21]. Moreover, in the superlattice of graphene/hBN, measured dI/dV curves show evident dips, and it is interpreted as a result of the emergence of a new set of Dirac points due to the superlattice potential [21,36,49]. However, the influence of hBN substrates on graphene under various twisting angles is yet not fully understood.

In this paper, we will study graphene/hBN systems by first-principles calculations to address effects of the periodic potential induced by moiré patterns. The joint action of global symmetry breaking and the local stacking order in the moiré pattern will be discussed. The weak interlayer van der Waals interaction was usually believed to have little influence on graphene and was deemed as no more than a mechanical supporting substrate. In our study, it is found that, although the basic properties of graphene are preserved, the BN sublayer has salient effects on graphene, such as charge transfer, charge redistribution, band shift, and energy gap modification, which will be detailed in the following section. As it is known, the size of the observed graphene/hBN moiré structure is too large for normal density functional theory (DFT) calculations. Therefore, the aim of our DFT calculations is to study the evolution trends of the gap, Fermi velocity, and density of states (DOS) with respect to the twisting angle, rather than to investigate properties that are only manifested in very-large-sized Gr/BN systems. Simultaneously, we also attempt to give a more unified understanding of the change of the Fermi velocity, bands, and DOS based on charge redistribution and transfer induced by interlayer interactions.

## 2. Results and Discussion

In the following section, BN always refers to hexagonal BN. The graphene/hBN system is briefed as Gr/BN. Gr/BN-θ denotes that graphene and BN have a relative twisting angle of θ. In Gr/BN, graphene is unstrained, whereas the lattice of BN is adapted to match with graphene. The experimental lattice constants of graphene and boron nitride are 2.460 and 2.504 Å, respectively. In reality, the moiré pattern formed in Gr/BN is owing to either the primitive lattice mismatch or the twisting angle or both. In the following section, these two factors are first discussed separately and then considered simultaneously. In our study of the Gr/BN moiré super lattice, the graphene super lattice is kept unstrained.

### 2.1. Untwisted Gr/BN Moiré Systems

**Figure 1:**
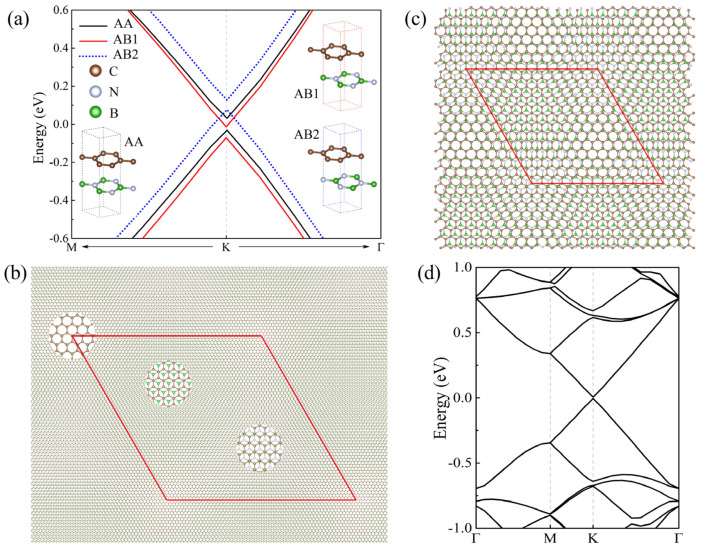
(**a**) Energy bands around the Dirac point K of Gr(1 × 1)/BN(1 × 1) with AA, AB1, and AB2 stacking. Their energy gaps are located at different positions. (**b**) The top view of untwisted Gr(56 × 56)/BN(55 × 55). Three circled regions give the zoomed view of the local stacking, which is quasi-AA, AB2, and AB1 from left to right, respectively. (**c**) The top view of the atomic structure of Gr(13 × 13)/BN(12 × 12). Central regions of the upper left and lower right of the diamond have the quasi-AB2 and -AA stacking. Regions near four vertices have the quasi-AB1 stacking. (**d**) The energy bands of Gr(13 × 13)/BN(12 × 12). The red diamond denotes the moiré superlattice.

We first consider the untwisted Gr/BN system without lattice mismatch. For Gr(1 × 1)/BN(1 × 1) systems, the energy gap is found to be 62.5 meV for AA-stacked Gr/BN, whereas it is reduced to 57.6 (50.5) meV for AB1 (AB2) stacking with half of the C atoms facing the B (N) atoms, as shown in Figure 1a. The gap opening is due to the breaking of the symmetry of graphene induced by BN. In AA stacking, all C atoms are right above the B and N atoms, giving rise to larger interlayer interaction and hence the larger band gap. Although normal DFT calculations usually underestimate the band gap, it can describe the gap variation trend in different systems.

In untwisted Gr/BN systems, there is interlayer in-commensuration due to the lattice mismatch. The number of C atoms per unit area is not equal to that of B and N atoms, and therefore the interlayer atomic registry becomes disordered in comparison with that of the perfect AA- or AB-stacked Gr/BN. The realistic untwisted system is Gr(56 × 56)/BN(55 × 55) with a periodicity of about 14 nm. It contains 12,322 atoms, which is well beyond the reach of the normal density functional theory method. Close examination found that Gr(56 × 56)/BN(55 × 55) features local quasi-AA and -AB stackings, as shown in Figure 1b. To simulate the effect of the quasi-AA and -AB stackings in mismatched systems, we calculated the Gr(13 × 13)/BN(12 × 12) model moiré system. Apparently, as shown in Figure 1c, Gr(13 × 13)/BN(12 × 12) with disordered stacking is much more asymmetric than AA- or AB-stacked Gr(1 × 1)/BN(1 × 1). One would expect that the energy gap of the former should be larger than that of the latter. The band structure is calculated as shown in Figure 1d. It is found that the Dirac cone is almost intact, with the energy gap being about 9.7 meV, which is much smaller than that of the AA- and AB-stacked Gr(1 × 1)/BN(1 × 1), in contradiction to the argument of symmetry.

In Figure 1c, the local stacking in some region of Gr(13 × 13)/BN(12 × 12) is quasi-AA stacking, and in some other region is quasi-AB stacking, reproducing local stacking features of Gr(56 × 56)/BN(55 × 55). Although the AA- and AB-stacked Gr(1 × 1)/BN(1 × 1) are appreciably gapped, their energy gap positions are different. As seen in Figure 1a, the gap of AB2 stacking is above those of AA and AB1 stacking, whereas the gaps of the latter overlap. If the three stackings coexist, the resulting band structure should be much less gapped. Therefore, for Gr/BN systems with both local quasi-AA and -AB stackings, which is the case for Gr(13 × 13)/BN(12 × 12) shown in Figure 1c, the overall energy gap is much smaller than that of Gr(1 × 1)/BN(1 × 1) with solely AA or AB stacking. In experiments [37], it was found that the moiré Gr/BN is almost gapless.

In Gr(13 × 13)/BN(12 × 12), although BN is stretched by about 6.4%, which seems to be fairly large, it can be served as a model system to represent a system with local quasi-AA and -AB stacking. To be more realistic, we also calculated the untwisted Gr(28 × 28)/BN(27 × 27) system with up to 3026 atoms using DFTB+ [50], in which the tensile strain in BN has been reduced to 1.9%. The geometrical feature of the coexistence of local quasi-AA and -AB stacking regions remains (Supplementary Materials Figure S6). The calculated band gap is 0.4 meV as the result of the overlapping of the gaps of different stacking regions, as discussed above.

### 2.2. Twisted Gr/BN Moiré Systems

Since the lattice mismatch between the primitive lattices of graphene and BN is smaller than 1.8%, it is usually assumed that the two layers take the same primitive lattice constant (2.460 Å) in the twisted Gr/BN-θ systems [49], which will introduce about 1.8% compressive strain in the BN layer. We first neglect the lattice mismatch in the twisted moiré systems, and then we consider both the lattice misalignment and the primitive lattice mismatch simultaneously. It will be seen that the evolution of the Fermi velocity, the dip in the density of states, and the charge transfer with respect to angles (the periodicity of the moiré structure) are almost not affected by the small primitive lattice mismatch.

Following Ref. [51], we considered the twisting angles of 21.78°, 13.1°, 9.43°, 7.34°, 5.1°, and 3.48° to obtain the moiré superlattices, in which the primitive lattice mismatch in the graphene and BN layers is neglected (both taking the same primitive lattice constant, 2.46 Å). The results for the different twisted systems are listed in Table 1. The calculated mean interlayer distance is about 3.39 Å and almost does not vary with the twisting angles. Graphene remains almost flat, since the buckling of C atoms induced by the BN substrate is very small. The energy gap is as small as several meV. With the decrease in the twisting angle, the energy gap has a general decreasing trend.

**Figure 2:**
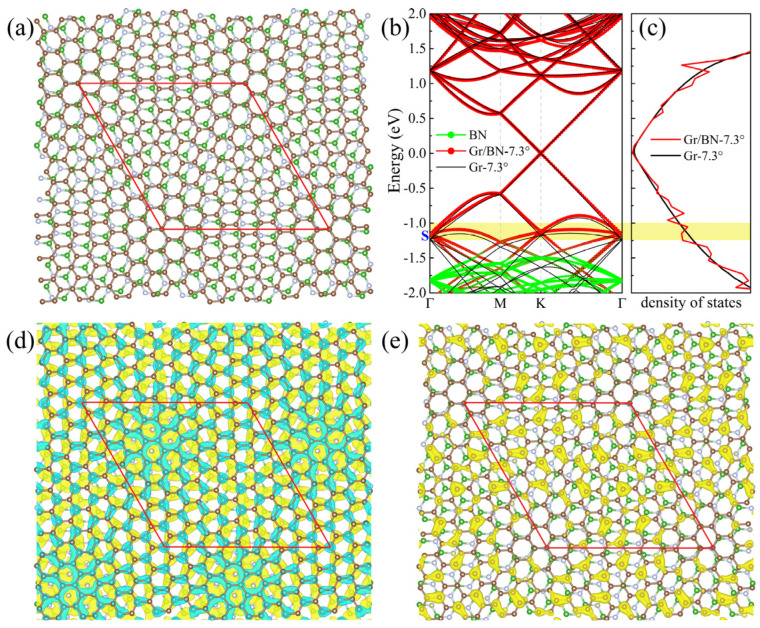
(**a**)The top view of the atomic structure of Gr/BN-7.34°. Brown, green, and grey spheres denote C, B, and N atoms, respectively. (**b**) The band structure of Gr/BN-7.34° is in red dots, and that of Gr-7.34° is in black lines. Green dots denote the contribution of BN layer. (**c**) The DOS of Gr/BN-7.34° is in red lines, and that of perfect graphene is in black line, calculated on a Γ-centered 15 × 15 × 1 k-mesh. (**d**) The differential total charge density. The green color indicates loss of electrons. (**e**) The charge density of the state S is labeled in (**b**). The red diamond denotes the moiré superlattice. Central regions of the upper left and lower right of the diamond have the quasi-AB2 and -AB1 stacking, respectively. Regions near four vertices have the quasi-AA stacking.

In the twisted Gr/BN-θ, graphene basically maintains its intrinsic lattice periodicity, and, at the same time, it is modulated by an external periodic potential imposed by BN. Below, we will focus on the Gr/BN-7.34° system, as depicted in Figure 2a, to discuss the properties of the twisted Gr/BN-θ. Without losing generality, the discussion applies to other twisting angles whose band structure, DOS, charge distribution, and charge transfer are listed in Supplemental Materials (Figures S1–S5).

BN has a large energy gap, and its band edge is far from the Dirac point of graphene. Therefore, the Dirac cones of graphene falling within the gap of BN are almost not affected by BN (Figure 2b). The calculated charge density of Gr/BN at the Dirac point is almost the same as that of the perfect graphene. In Figure 2a, it can be seen that there are also quasi-AB and -AA stacking regions in Gr/BN-7.34°. As discussed in Section 2.1, the energy gap is much smaller than that of the AB- and AA-stacked Gr(1 × 1)/BN(1 × 1).

**Figure 3:**
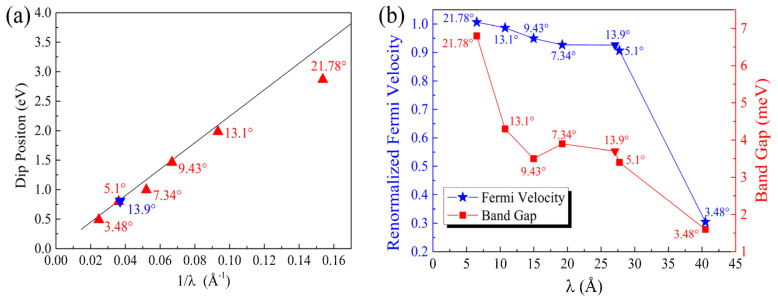
(**a**) The dip position *E*_dip_ in the DOS with respect to the Fermi level and (**b**) the Fermi velocity and band gap at the Dirac point vs the super lattice constant λ of Gr/BN-θ. The solid line in (**a**) denotes the theoretical trend  $E_{dip}=2\pi \hbar v_{F}/\sqrt{3}\lambda$ [36]. The corresponding twisting angles are labeled. The inverted triangle denotes the data point of the nearly perfectly matched system of Gr/BN-13.90°.

We calculated the normalized Fermi velocity of the twisted Gr/BN, $v_{f}\left(Gr/BN\right)/v_{f}\left(Gr\right)$, where $v_{f}\left(Gr/BN\right)$ and $v_{f}\left(Gr\right)$ are the Fermi velocities of Gr/BN and the perfect graphene, respectively. As shown in Figure 3a, it decreases with the decrease in the twisting angle. The reduction of the Fermi velocity is considerable, especially at 3.48°. The Dirac cone of graphene is formed by the interaction of the p_z_ states of C atoms. The strength of the C_p_z_ state interaction determines the slope of the Dirac cone. The larger the interaction strength, the larger is the slope, which is proportional to the Fermi velocity $v_{f}=-\partial E/\hbar \partial k$. We then calculated the charge transfer between graphene and BN to illustrate the variation of the Fermi velocity. It can be seen that the p_z_ states of the C atoms lost electrons (Figure 2d), and hence the interaction between them is reduced, lowering down the Fermi velocity. It has been found that the in-plane electric field can reduce the Fermi velocity considerably [52]. As the charge transfer is uneven in the graphene plane (Figure 2d), an in-plane electric field will be induced, which will further reduce the Fermi velocity. As the size of the moiré lattice increases with the decrease in the twisting angle, the range of the net in-plane electric field increases, giving rise to lower Fermi velocity. The electronegativity of N atoms is larger than that of C atoms. It is found that the C atoms right above lose more electrons.

The periodic potential imposed by the underlying BN layer will modify the band structure of graphene. For comparison, we also calculated the Gr-7.34° by removing the BN underlayer from the Gr/BN7.34° system. It can be seen in Figure 2b that between −0.5 and 0.0 eV, the bands of Gr-7.34° and Gr/BN-7.34° almost coincide. The bands between −0.5 and −1.2 eV almost entirely come from C atoms, with nearly zero contribution from the B and N atoms, which seems to suggest that the bands of Gr-7.34° and Gr/BN-7.34° should also coincide in this energy range. Their valence bands are found to have similar dispersion. Closer examination can find that the bands turn flatter in Gr/BN-7.34° due to the interaction with BN. The band crossing points of Gr/BN-7.34° at Γ is pushed up by BN, leading to an up-shift of the bands. We calculated the charge density of the state S, as shown in Figure 2e. As expected, that charge is concentrated on the C layer. However, the charge distribution has been considerably modulated by the periodic potential exerted by BN, in contrast to the even distribution of the corresponding states of Gr-7.34°. The appreciably inhomogeneous charge distribution will raise the repulsive energy of the electrons, and hence S is pushed up. The characteristic length of the charge inhomogeneity is about the moiré lattice size, which increases with the decrease in the twisting angle, and, as a result, the S state is more pushed up in energy.

It was observed experimentally that there are dips in the dI/dV curves of twisted Gr/BN, which are absent in those of ideal graphene. The dips approach the Fermi energy as the twisting angle decreases, since dI/dV is proportional to the DOS. As an illustration, the calculated DOS of Gr/BN-7.34° is shown in Figure 2c. The position of the dip in the valence band is plotted in Figure 3a. It is found that the theoretical dip position with respect to the Fermi level is inversely proportional to the super lattice constant λ, $E_{dip}=2\pi \hbar v_{F}/\sqrt{3}\lambda$ [36], which is depicted as the solid line in Figure 3a. The fitting value of $v_{F}$ is 0.94 × 10^−6^ m s^−1^. The calculated DOS dip approaches the Fermi energy as the super lattice constant increases, following the theoretical trend well.

In Figure 2b,c, it can be seen that the dip position corresponds to the S state. The upshift of the S state will reduce the dispersion of the bands between the S state and the Dirac point state. The inhomogeneity of the S state will contribute to the charge transfer, which will affect the Fermi velocity. Therefore, the change of the states around S correlates the change of the DOS and the Fermi velocity. As discussed above, the upshift of the S state with the decrease in the twisting angle leads to the shift of the dip in the DOS towards the Fermi energy. From Figure 3b, it can be seen that the band gap at the Dirac point also decreases with the increase in the super lattice constant.

**Figure 4:**
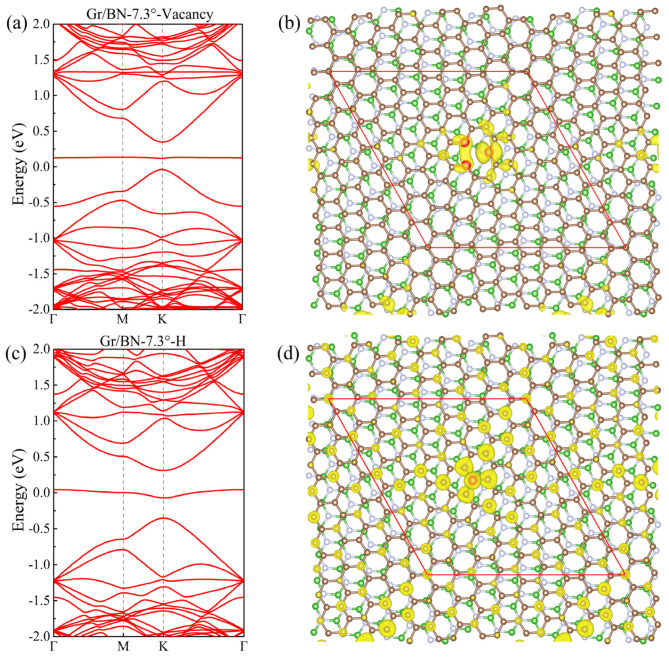
(**a**) The band structure of Gr/BN-7.34° with a vacancy. (**b**) The charge distribution of the flat band within the gap of (**a**). (**c**,**d**) corresponding to (**a**,**b**), respectively, for Gr/BN-7.34° with an adsorbed H atom. The C atoms around the vacancy in (**b**) and the adsorbed H atom in (**d**) are distinguished as red spheres.

To study the influence of defects, we studied Gr/BN-7.34° with vacancy and adsorbed H, respectively, as shown in Figure 4. It is found that a sizable gap is opened at the Dirac point, and a flat band, contributed by the defect, passes through the gap. The charge distribution of the flat band is concentrated around the defects. It can be expected that with an increase in the size of the moiré structure, the concentration of the defects will drop, and the gap at the Dirac point will decrease.

**Figure 5:**
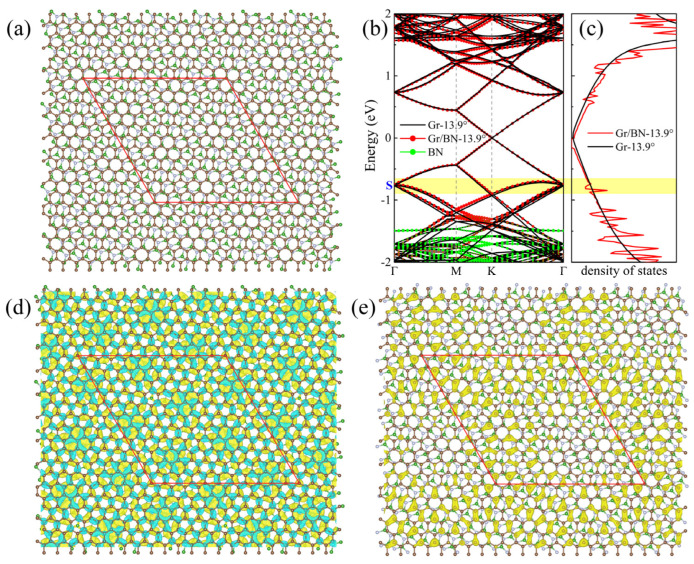
(**a**) The top view of the atomic structure of the Gr/BN-13.90° nearly perfect superlattice match, taking into account the primitive lattice mismatch and the twisting angle. The brown, green, and grey spheres denote C, B, and N atoms, respectively. (**b**) The band structure of Gr/BN-13.90° is in red dots, and that of Gr-13.90° is in black lines. Green dots denote the contribution of BN layer. (**c**) The DOS of Gr/BN-13.90° is in red lines, and that of perfect graphene is in black lines, calculated on a Γ-centered 11 × 11 × 1 k-mesh. (**d**) The differential total charge density. The green color indicates loss of electrons. (**e**) The charge density of the state S is labeled in (**b**). The red diamond denotes the moiré superlattice.

In the above-studied twisting systems, the neglect of the primitive lattice mismatch between graphene and BN introduces a superlattice strain (~1.8% strain in BN). To illustrate if this little strain will appreciably change the above-discussed evolution of the electronic structures of the twisting Gr/BN systems, we searched possible Gr/BN moiré systems via taking account of the primitive lattice mismatch and the rotation between the Gr and BN layers simultaneously. It is found that a Gr/BN-13.90° system has an almost-perfect superlattice match, in which the BN layer has a tiny strain of 0.09%, which is much smaller than 1.8%. There are 242 C atoms and 234 B and N atoms in Gr/BN-13.90°, and the superlattice constant is 27.06 Å, as shown in Figure 5a. The energy gap is fairly small as 0.37 meV. The calculated normalized Fermi velocity is 0.92. Corresponding to Figure 2, the above discussion about the electronic properties of Gr/BN-7.3°, such as the dip position in the DOS, the charge transfer, and redistribution, applies here to those of Gr/BN-13.90°, as shown in Figure 5. As can be seen in Figure 3, Gr/BN-13.90° and Gr/BN-5.1° have similar superlattice constants (~27 Å), and they both have a similar band gap, Fermi velocity, and dip position in the DOS, indicating that the electronic properties of the nearly perfectly matched Gr/BN-13.90° (with nearly zero strain in the BN layer) follow the same evolution lines shown in Figure 3.

We also found a nearly perfectly matched system, Gr/BN-1.95°, in which the BN layer has a tiny strain of 0.11%. The super lattice constant is 63.96 Å, containing 2654 atoms in total, with 1352 C and 1302 B and N atoms. Its electronic properties are calculated by DFTB+ (Supplementary Materials Figure S7). The band gap at the Dirac point is 0.51 meV. It can be seen that the dip position in the DOS is 0.37 eV, which agrees well with the theoretical value of 0.35 eV. The normalized Fermi velocity is found to be reduced to 0.44.

## 3. Methods

The first-principles calculation is performed within the framework of density functional theory using local density approximation, as implemented in the Vienna Ab-initio Simulation Package [53,54,55,56,57]. The electron-core interaction is treated by the projector augmented wave pseudopotential [58,59]. It has been tested that the energy cutoffs of 300 eV and 400 eV produce almost-indistinguishable results, and, therefore, the energy cutoff set to 400 eV in calculation will give good convergence. The tetrahedron method is used in the calculation of the DOS. The atoms are relaxed until the maximum force on each atom is smaller than 0.01 eV/Å. The supercell approach is adopted with a slab of graphene/BN and a vacuum of 10 Å.

## 4. Conclusions

In conclusion, we have made a computational study on the untwisted and twisted Gr/BN systems. The moiré pattern is formed as a result of a small lattice difference between graphene and BN, and the relative interlayer twisting. It is found that the Dirac cones of graphene are well preserved in the heterostructure, and the graphene overlayer remains almost flat. In the perfectly matched AA- and AB-stacked Gr/BN, there is a sizable energy gap of several tens of meV, but the gaps have a staggered alignment. Therefore, in the moiré Gr/BN with both quasi-AA and -AB stacked regions, the overall gap (only several meV) is almost closed. There is an inhomogeneous electron transfer from graphene to BN, reducing the interaction of the C_p_z_ states and inducing an in-plane electric field in graphene, which will give rise to an appreciable reduction of the Fermi velocity. The periodic potential imposed by BN in the moiré Gr/BN modulates the charge distribution, resulting in the upshift of the valence bands of graphene. In comparison to the smooth DOS of perfect graphene, there are dips in the DOS of the twisted moiré Gr/BNs, which approaches the Fermi energy as the size of the moiré super lattice increases; this is in good agreement with the experiments. Depending on the high electronic qualities of semiconducting graphene, promising applications are expected in next-generation digital logic devices by enhancing the energy gap in twisted Gr/BN systems.

## Figures and Tables

**Table 1:** The C atom buckling, band gap, number of atoms, interlayer distance between graphene and BN, and lattice constants of the twisted Gr/BN-θ moiré superlattices.

Angle (°)	21.78	13.1	9.43	7.3	5.1	3.48
C atom buckling (Å)	0.0024	0.020	0.017	0.093	0.068	0.017
Band Gap (meV)	6.8	4.3	3.5	3.9	3.4	1.6
Number of atoms	28	76	148	244	508	1084
Gr-BN distance (Å)	3.399	3.396	3.377	3.394	3.396	3.400
Latt. Const. (Å)	6.509	10.723	14.964	19.213	27.723	40.497

## Data Availability

The datasets used and/or analyzed during the current study are available from the corresponding author on reasonable request.

## Supplementary Materials

The supporting information can be downloaded at: https://www.mdpi.com/article/10.3390/molecules27123740/s1.
