# Supplementary material for: Influence of Hexagonal Boron Nitride on Electronic Structure of Graphene
Source: Molecules. 2022 Jun 10;27(12):3740. doi: 10.3390/molecules27123740 (PMC9227148; doi:10.3390/molecules27123740)
Supplement: Supplementary file 1 [file molecules-27-03740-s001.zip › molecules-1695883-supplementary.pdf]

Supplementary Materials for
Influence of hexagonal boron nitride on electronic structure of graphene

Jingran Liu [1], Chaobo Luo [1], Haolin Lu [2], Zhongkai Huang [3,*], Guankui Long [2,*], and Xiangyang Peng [1,*]

[1] Hunan Key Laboratory for Micro-Nano Energy Materials and Devices, School of Physics and Optoelectronics, Xiangtan University, Hunan 411105, China; 201921001241@smail.xtu.edu.cn (J.L.); luochaobo@xtu.edu.cn (C.L.)

[2] School of Materials Science and Engineering, National Institute for Advanced Materials, Renewable Energy Conversion and Storage Center (RECAST), Nankai University, 300350, Tianjin, China; 1713638@mail.nankai.edu.cn (H.L.)

[3] Key Laboratory of Extraordinary Bond Engineering and Advanced Materials Technology of Chongqing, Yangtze Normal University, Chongqing 408100, China;

* Correspondence: zhongkaihuang@yznu.edu.cn (Z.H.); longgk09@nankai.edu.cn (G.L.); xiangyang_peng@xtu.edu.cn (X.P.);

Contents:

1. The supplementary results calculated by VASP neglecting primitive lattice mismatch
The twisted Gr/BN-θ systems calculated by VASP, neglecting the mismatch between the primitive lattices of graphene and BN: Gr/BN-21.8°, Gr/BN-13.1°, Gr/BN-9.4°, Gr/BN-5.1°, and Gr/BN-3.48°.

2. The supplementary results calculated by DFTB+ considering primitive lattice mismatch
The Gr/BN systems calculated by DFTB+, including the mismatch between the primitive lattice of graphene and BN: the untwisted Gr(28×28)/BN(27×27) and the twisted Gr/BN-1.95° systems

References of the Supplementary Materials

1. The supplementary results calculated by VASP neglecting primitive lattice mismatch

Vienna Ab-initio Simulation Package (VASP) [1-5] is used to produce the detailed results for Gr/BN-21.8°, Gr/BN-13.1°, Gr/BN-9.4°, Gr/BN-5.1°, and Gr/BN-3.48°, shown in Figs. S1-S5, respectively. For discussion, please see the main text.

Gr/BN-21.8°

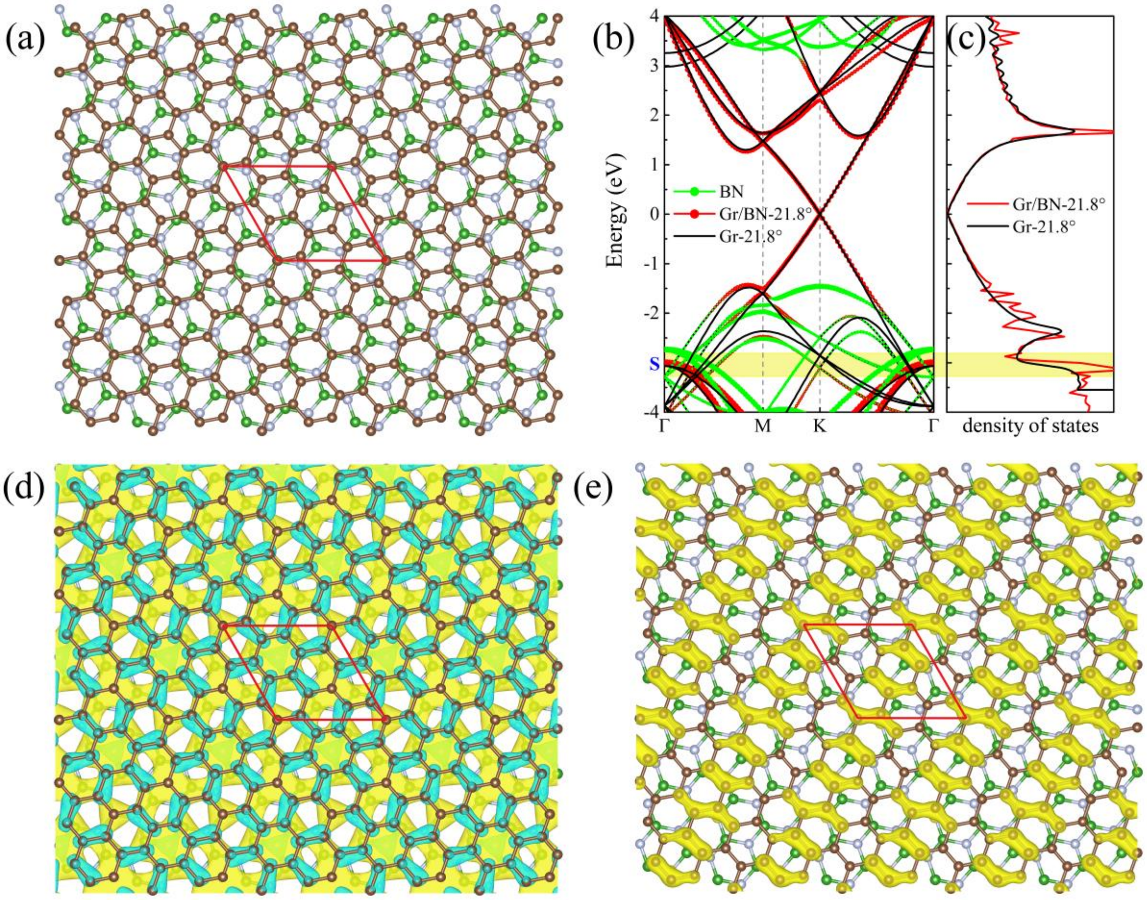

Figure S1. (a)Top view of the atomic structure of Gr/BN-21.8°. Brown, green and grey spheres denote C, B and N atoms, respectively. (b) Band structure of Gr/BN-21.8° in red dots and that of Gr-21.8° in black lines. Green dots denote contributions of BN layer. (c) DOS of Gr/BN-21.8° in red lines and that of perfect graphene in black line, calculated on a Γ-centered 31×31×1 k-mesh. (d) Differential total charge density. Green color indicates loss of electrons. (e) Charge density of the state with respect to an energy region marked by the yellow color bar in (b). Red diamond denotes a moiré superlattice induced by a 21.8° rotation between the upper and lower layers.

Gr/BN-13.1°

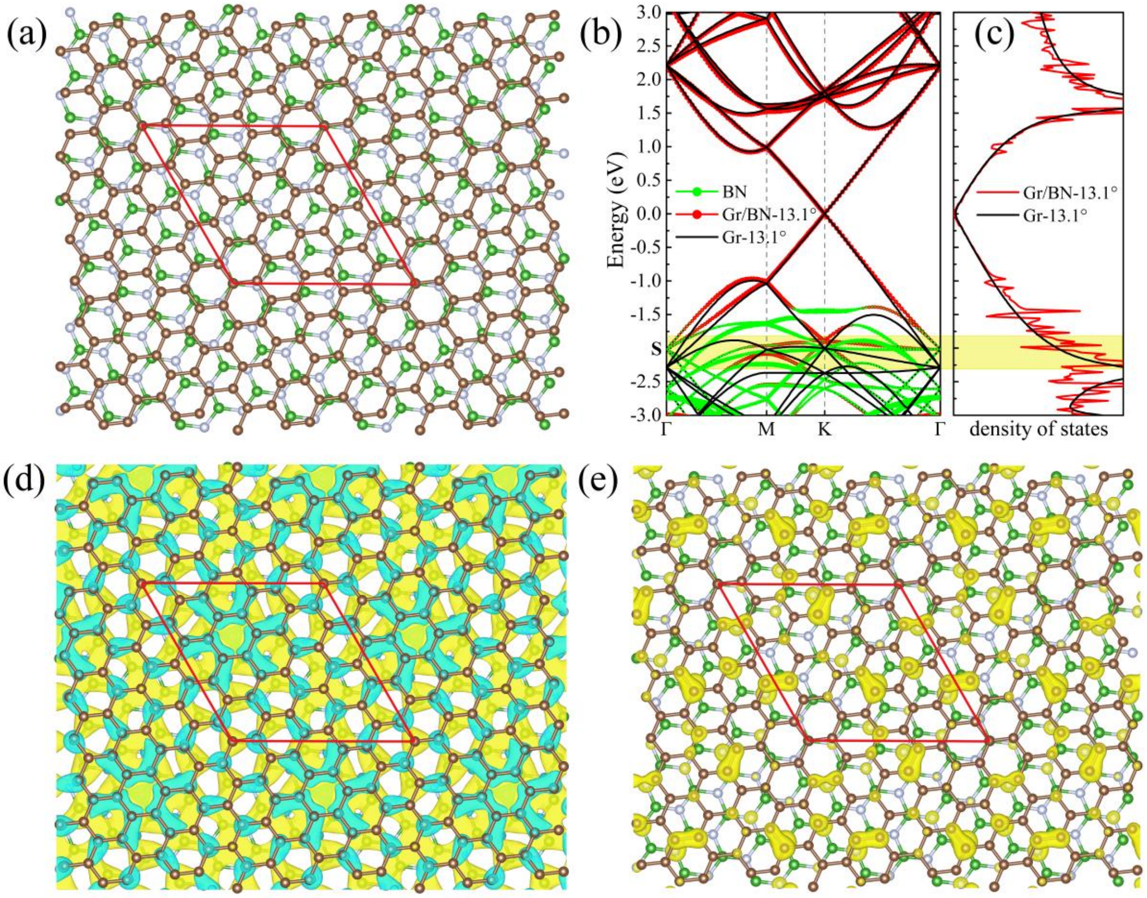

Figure S2. (a)Top view of the atomic structure of Gr/BN-13.1°. Brown, green and grey spheres denote C, B and N atoms, respectively. (b) Band structure of Gr/BN-13.1° in red dots and that of Gr-13.1° in black lines. Green dots denote contributions of BN layer. (c) DOS of Gr/BN-13.1° in red lines and that of perfect graphene in black line, calculated on a Γ-centered 21×21×1 k-mesh. (d) Differential total charge density. Green color indicates loss of electrons. (e) Charge density of the state with respect to an energy region marked by the yellow color bar in (b). Red diamond denotes a moiré superlattice induced by a 13.1° rotation between the upper and lower layers.

Gr/BN-9.4°

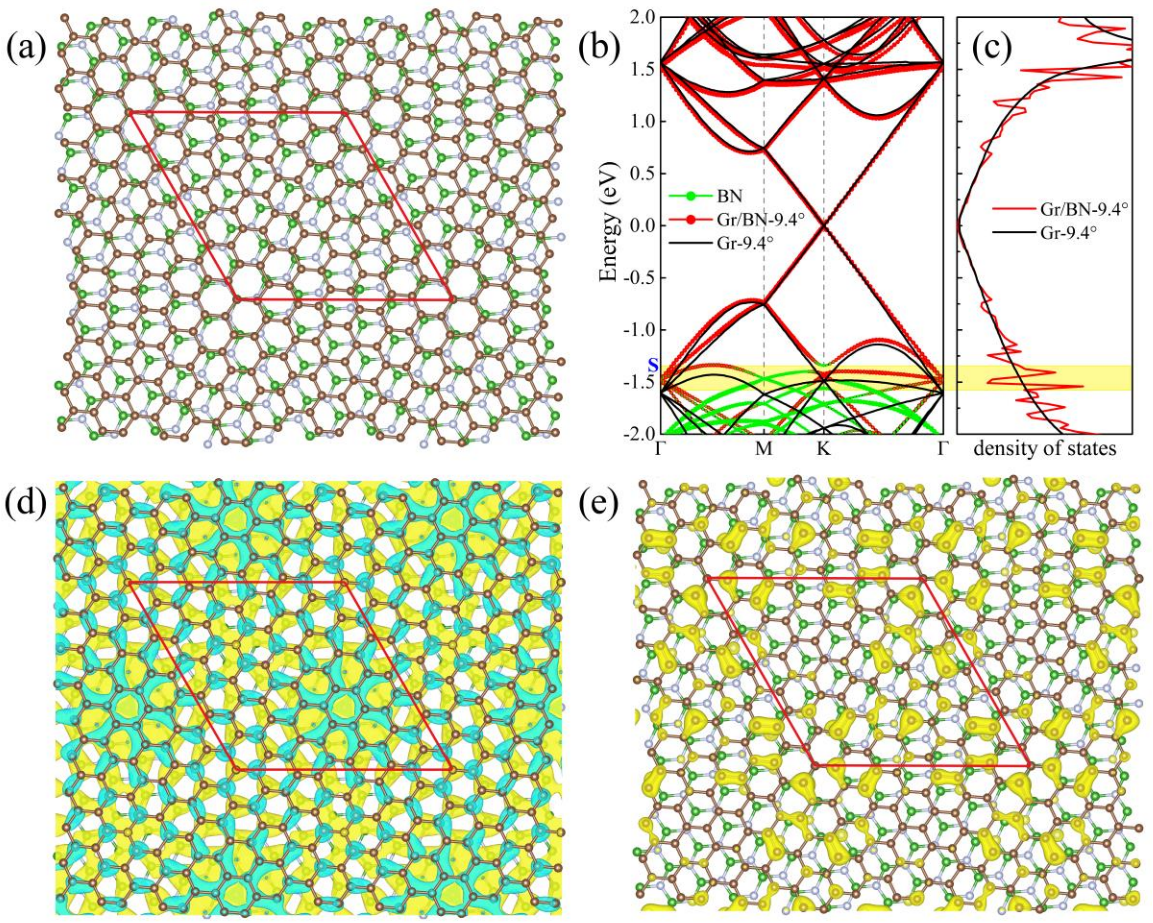

Figure S3. (a)Top view of the atomic structure of Gr/BN-9.4°. Brown, green and grey spheres denote C, B and N atoms, respectively. (b) Band structure of Gr/BN-9.4° in red dots and that of Gr-9.4° in black lines. Green dots denote contributions of BN layer. (c) DOS of Gr/BN-9.4° in red lines and that of perfect graphene in black line, calculated on a Γ-centered15×15×1 k-mesh. (d) Differential total charge density. Green color indicates loss of electrons. (e) Charge density of the state with respect to an energy region marked by the yellow color bar in (b). Red diamond denotes a moiré superlattice induced by a 9.4° rotation between the upper and lower layers.

Gr/BN-5.1°

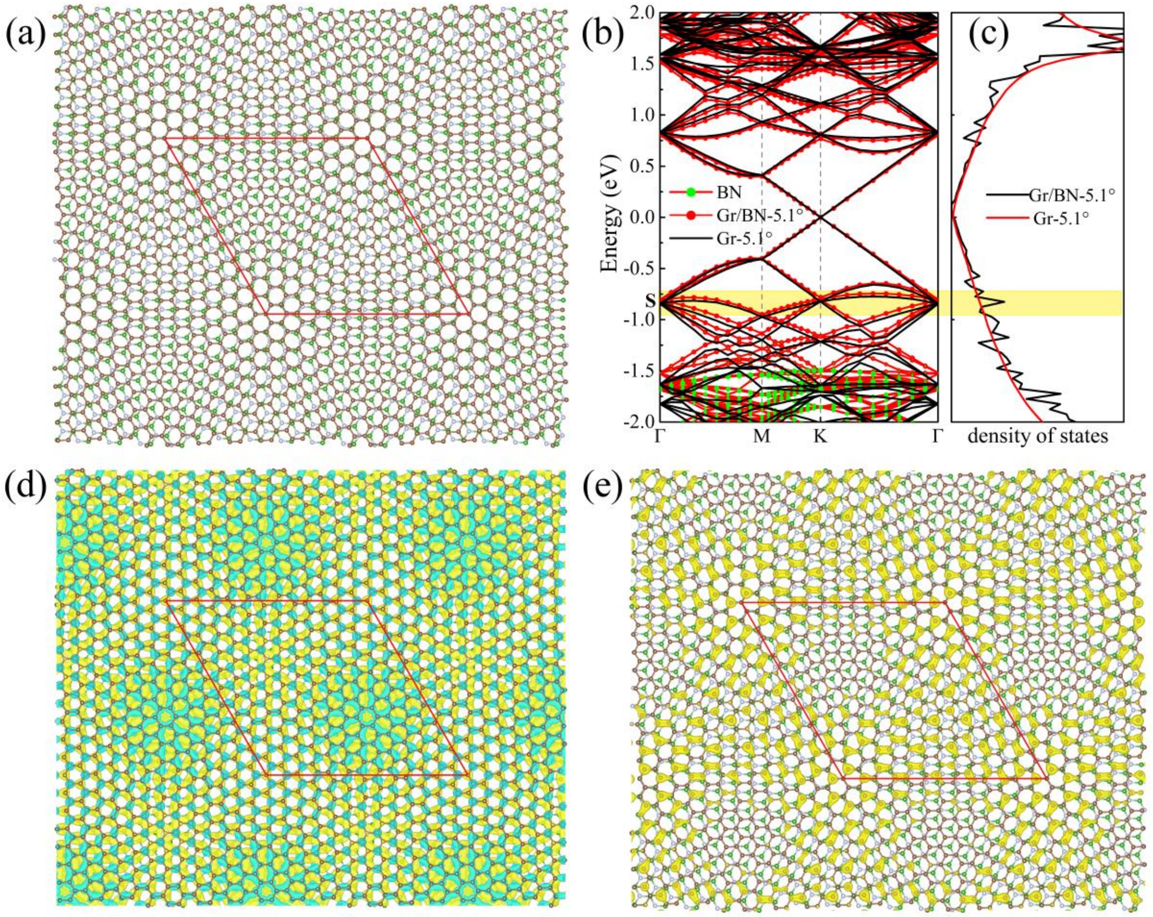

Figure S4. (a)Top view of the atomic structure of Gr/BN-5.1°. Brown, green and grey spheres denote C, B and N atoms, respectively. (b) Band structure of Gr/BN-5.1° in red dots and that of Gr-5.1° in black lines. Green dots denote contributions of BN layer. (c) DOS of Gr/BN-5.1° in red lines and that of perfect graphene in black line, calculated on a Γ-centered11×11×1 k-mesh. (d) Differential total charge density. Green color indicates loss of electrons. (e) Charge density of the state with respect to an energy region marked by the yellow color bar in (b). Red diamond denotes a moiré superlattice induced by a 5.1° rotation between the upper and lower layers.

Gr/BN-3.48°

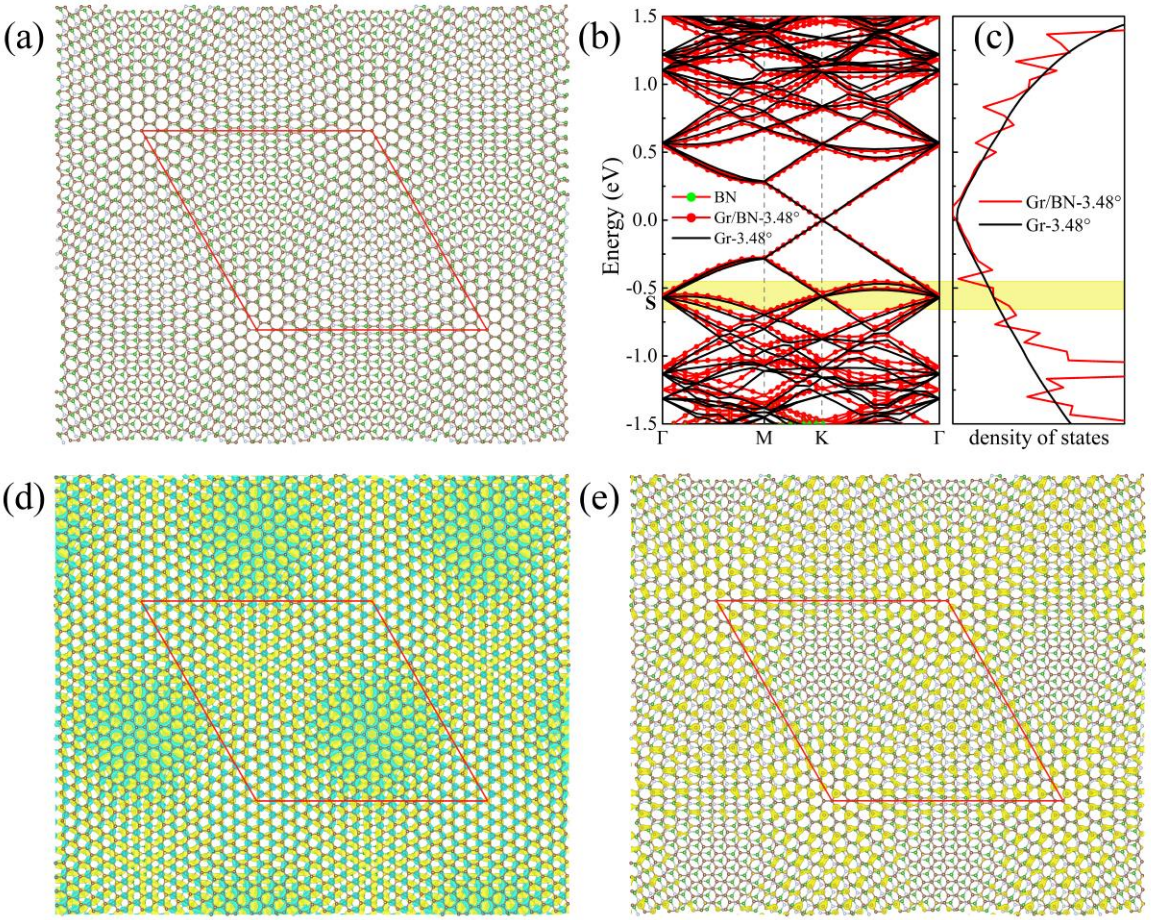

Figure S5. (a)Top view of the atomic structure of Gr/BN-3.48°. Brown, green and grey spheres denote C, B and N atoms, respectively. (b) Band structure of Gr/BN-3.48° in red dots and that of Gr-3.48° in black lines. Green dots denote contributions of BN layer. (c) DOS of Gr/BN-3.48° in red lines and that of perfect graphene in black line, calculated on a Γ-centered 6×6×1 k-mesh. (d) Differential total charge density. Green color indicates loss of electrons. (e) Charge density of the state with respect to an energy region marked by the yellow color bar in (b). Red diamond denotes a moiré superlattice induced by a 3.48° rotation between the upper and lower layers.

2. The supplementary results calculated by DFTB+ considering primitive lattice mismatch

The following calculated Gr/BN systems are very large, containing 3026 and 2654 atoms, respectively. The computational cost is increasing dramatically if the atom number grows larger. Comparing with VASP [1-4,6,7], the Density Functional based Tight Binding (DFTB+) model [8] is computationally affordable for large sized Gr/BN moiré patterns. The matsci-0-3 DFTB basis set is utilized in construction of the Hamiltonians [9]. For discussion of the figures below, please see the main text.

The untwisted Gr(28×28)/BN(27×27)

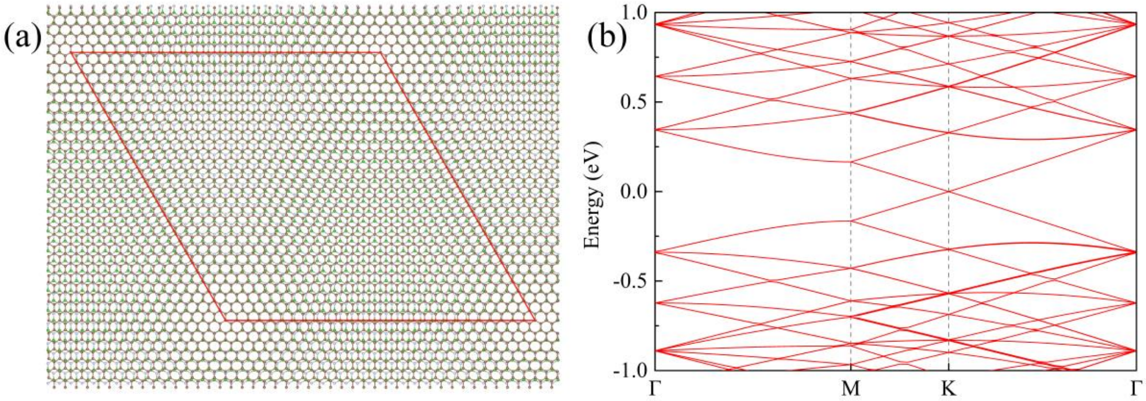

Figure S6. (a) the top view of the atomic structure of the untwisted Gr(28×28)/BN(27×27). Central regions of the upper left and lower right of the diamond have the quasi AB1 and AB2 stacking. Regions near the four vertices have the quasi-AA stacking. The red diamond denotes the moiré superlattice. (b) The energy bands of Gr(28×28)/BN(27×27).

Gr/BN-1.95° simultaneously considering the primitive lattice match and interlayer twisting

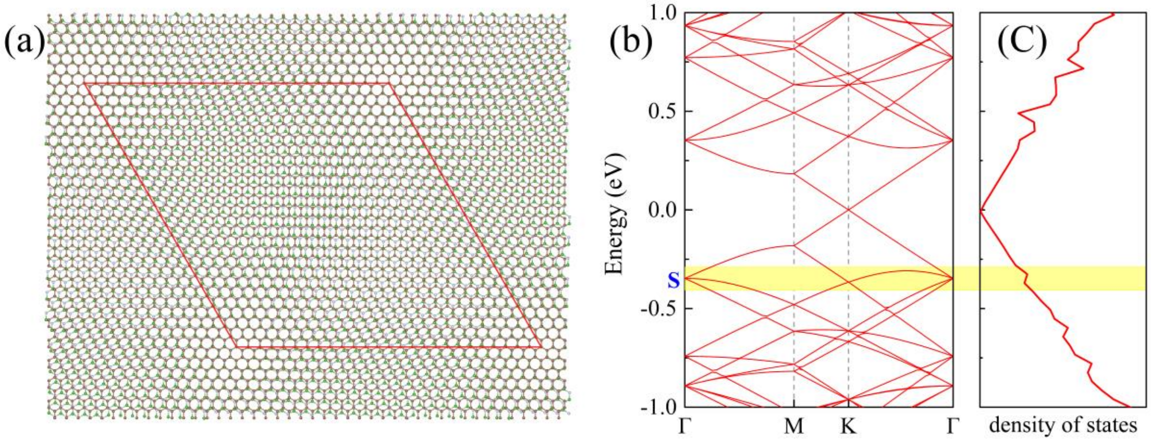

Figure S7. (a)Top view of the atomic structure of Gr/BN-1.95°, in which the super lattice constants of the unstrained graphene and BN are 63.96 and 63.89 Å, respectively, resulting a tiny super lattice mismatch of 0.11%. Brown, green and grey spheres denote C, B and N atoms, respectively. Red diamond denotes a moiré superlattice induced by a 1.95° rotation between the upper and lower layers. (b) The band structure of Gr/BN-1.95°. (c) The DOS of Gr/BN-1.95°, calculated on a Γ-centered 21×21×1 k-mesh. The first dip in DOS below fermi-level is marked by a yellow color bar, and its energy position correspond well to the S position in (a).

References for Supplementary Materials

1. Blöchl, P.E. Projector augmented-wave method. *Physical Review B* **1994**, *50*, 17953-17979, doi:10.1103/PhysRevB.50.17953.
2. Kresse, G.; Joubert, D. From ultrasoft pseudopotentials to the projector augmented-wave method. *Physical Review B* **1999**, *59*, 1758-1775, doi:10.1103/PhysRevB.59.1758.
3. Hammer, B.; Hansen, L.B.; N?Rskov, J.K. Improved adsorption energetics within density-functional theory using revised Perdew-Burke-Ernzerhof functionals. *Physical Review B* **1999**, *59*, 7413-7421, doi:10.1103/PhysRevB.59.7413.
4. Perdew, J.P.; Chevary, J.A.; Vosko, S.H.; Jackson, K.A.; Pederson, M.R.; Singh, D.J.; Fiolhais, C. Erratum: Atoms, molecules, solids, and surfaces: Applications of the generalized gradient approximation for exchange and correlation. *Physical Review B* **1993**, *48*, 4978-4978, doi:10.1103/PhysRevB.48.4978.2.
5. Monkhorst, H.J.; Pack, J.D. Special points for Brillouin-zone integrations. *Physical review B* **1976**, *13*, 5188, doi:10.1103/PhysRevB.13.5188.
6. Kresse, G.; Furthmüller, J. Efficient iterative schemes for ab initio total-energy calculations using a plane-wave basis set. *Physical review B* **1996**, *54*, 11169, doi:10.1103/PhysRevB.54.11169.
7. Kresse, G.; Furthmüller, J. Efficiency of ab-initio total energy calculations for metals and semiconductors using a plane-wave basis set. *Computational materials science* **1996**, *6*, 15-50, doi:10.1016/0927-0256(96)00008-0.
8. Hourahine, B.; Aradi, B.; Blum, V.; Bonafé, F.; Buccheri, A.; Camacho, C.; Cevallos, C.; Deshaye, M.Y.; Dumitrică, T.; Dominguez, A.; et al. DFTB+, a software package for efficient approximate density functional theory based atomistic simulations. *The Journal of Chemical Physics* **2020**, *152*, 124101, doi:10.1063/1.5143190.
9. Lukose, B.; Kuc, A.; Frenzel, J.; Heine, T. On the reticular construction concept of covalent organic frameworks. *Beilstein J Nanotechnol* **2010**, *1*, 60-70, doi:10.3762/bjnano.1.8.
